# Two virulent sRNAs identified by genomic sequencing target the type III secretion system in rice bacterial blight pathogen

**DOI:** 10.1186/s12870-018-1470-7

**Published:** 2018-10-16

**Authors:** Yiqun Hu, Liyuan Zhang, Xuan Wang, Fengli Sun, Xiangxin Kong, Hansong Dong, Heng Xu

**Affiliations:** 1 Department of Plant Pathology, College of Plant Protection, Nanjing Agricultural University, Nanjing, 210095 Jiangsu Province China; 2 State Ministry of Education Key Laboratory of Integrated Management of Crop Pathogens and Insect Pests, Nanjing, 210095 Jiangsu Province China; 3 Current Address: Rural Work Bureau of Zhangpu Town, Suzhou, 215300 Jiangsu Province China

**Keywords:** *Xanthomonas oryzae* pv. *oryzae* (*Xoo*), Virulence, sRNA, Trans217, Trans3287, Type III secretion system (T3SS), PthXo1

## Abstract

**Background:**

Small non-coding RNA (sRNA) short sequences regulate various biological processes in all organisms, including bacteria that are animal or plant pathogens. Virulent or pathogenicity-associated sRNAs have been increasingly elucidated in animal pathogens but little is known about similar category of sRNAs in plant-pathogenic bacteria. This is particularly true regarding rice bacterial blight pathogen *Xanthomonas oryzae* pathovar *oryzae* (*Xoo*) as studies on the virulent role of *Xoo* sRNAs is very limited at present.

**Results:**

The number and genomic distribution of sRNAs in *Xoo* were determined by bioinformatics analysis based on high throughput sequencing (sRNA-Seq) of the bacterial cultures from virulence-inducing and standard growth media, respectively. A total of 601 sRNAs were identified in the *Xoo* genome and ten virulent sRNA candidates were screened out based on significant differences of their expression levels between the culture conditions. In addition, trans3287 and trans3288 were also selected as candidates due to high expression levels in both media. The differential expression of 12 sRNAs evidenced by the sRNA-Seq data was confirmed by a convincing quantitative method. Based on genetic analysis of *Xoo* ΔsRNA mutants generated by deletion of the 12 single sRNAs, trans217 and trans3287 were characterized as virulent sRNAs. They are essential not only for the formation of bacterial blight in a susceptible rice variety Nipponbare but also for the induction of hypersensitive response (HR) in nonhost plant tobacco. *Xoo* Δtrans217 and Δtrans3287 mutants fail to induce bacterial blight in Nipponbare and also fail to induce the HR in tobacco, whereas, genetic complementation restores both mutants to the wild type in the virulent performance and HR induction. Similar effects of gene knockout and complementation were found in the expression of *hrpG* and *hrpX* genes, which encode regulatory proteins of the type III secretion system. Consistently, secretion of a type III effector, PthXo1, is blocked in Δtrans217 or Δtrans3287 bacterial cultures but retrieved by genetic complementation to both mutants.

**Conclusions:**

The genetic analysis characterizes trans217 and trans3287 as pathogenicity-associated sRNAs essential for the bacterial virulence on the susceptible rice variety and for the HR elicitation in the nonhost plant. The molecular evidence suggests that both virulent sRNAs regulate the bacterial virulence by targeting the type III secretion system.

**Electronic supplementary material:**

The online version of this article (10.1186/s12870-018-1470-7) contains supplementary material, which is available to authorized users.

## Background

Small non-coding RNAs (sRNAs) are characteristic of unique nucleotide (nt) sequences comprising 50–500 nt under most circumstances and constitute a universal group of post-transcriptional regulators for gene expression [1–3]. They function by base pairing with target mRNAs via limited and extended complementarity, which are used to distinguish two categories, namely cis-sRNAs and trans-sRNAs, respectively [2]. In addition, many sRNAs require the RNA binding protein Hfq to maintain stability and co-regulate target transcripts [4, 5]. Because of the primary function in post-transcription of target genes, sRNAs are able to regulate numerous bioprocesses in all living organisms [2, 3, 6]. In particular, animal and plant pathogens including bacteria employ certain sRNAs to regulate pathogenicity or virulence on their hosts [3, 7–9].

Animal-pathogenic bacteria in the *Escherichia* and *Salmonella* genera possess approximately a half of bacterial sRNAs as identified to date, but only a few have been associated with bacterial pathogenicity and/or virulence [10–12]. *E. coli* RyhB is a member of small antisense regulatory RNA family, with requirement for RNA chaperone Hfq to maintain stability and perform function [13–15]. RyhB works with the ferric uptake regulator to regulate cellular iron homeostasis, the *clbA* gene transcription, and the colibactin production [15]. These responses affect pathogenicity not only in *E. coli* [16], but also in *Shigella flexneri* [17], *S. dysenteriae* [18], and *Listeria monocytogenes* [19]. One more example is from the animal pathogen *S. typhimurium*, which has at least 280 sRNAs, as identified by deep sequencing of Hfq-bound transcripts [20]. The sRNA InvS controls the bacterial invasion by coordinating the production of PrgH, a type III secretion apparatus protein, and FimZ, a negative regulator of invasion-related gene expression [21]. In *Staphylococcus aureus*, moreover, RNAIII is a pathogenicity-associated sRNA reported as the first case [22, 25] and a secreted effector protein with multiple functions in the bacterial virulence [22–24]. While RNAIII directly targets *hla*, *spa*, and *rot* genes involved in quorum sensing [22, 24], its noncoding parts act as antisense RNAs to regulate translation and stability of related transcription factors, major virulence effectors, and cell wall metabolism enzymes [24, 25]. RNAIII also governs the expression of *SA1000* mRNA, which hypothetically encodes a fibrinogen binding protein indispensable for bacterial adhesion to host surfaces and invasion of host tissues [25]. These demonstrations suggest that bacterial RNAs use distinct mechanisms while cooperating with different functional partners to regulate pathogenicity and virulence.

In contrast to extensive studies on sRNAs in animal-pathogenic bacteria, little is known about plant pathogens regarding functions of sRNAs in relevance to virulence or pathogenicity [3]. Limited information comes from major group of plant-pathogenic bacteria, especially *Xanthomonas* species and pathovar (pv.), with some sRNAs already identified in *X. campestris* pv. *vesicatoria* (*Xcv*), *X. campestris* pv. *campestris* (*Xcc*), *X. oryzae* pv. *oryzae* (*Xoo*) [26–31]. sX12 and sX13 from *Xcv* are the earliest and best studied sRNAs in the bacterial genus. Both sRNAs contribute to the bacterial virulence on host plant pepper [28, 32]. Deletion of sX13 not only eliminates *Xcv* virulence but also impinges the type III secretion system (T3SS) [32], which is ubiquitously present in Gram-negative bacteria and functions to deliver effectors en route to the host cytoplasm [33]. Knockout of sX13 results in decreased expression of genes encoding structural protein HrcJ, regulators HrpG and HrpX, and translocator HrpF of the T3SS [32]. In essence, HrpF serves as a type III translocator indispensable for effector translocation from bacterial cells into the cytosol of plant cells, where effectors execute their pathological functions [34]. In agreement with the effect of sX13 on T3SS, sRNA-Xcc1 transcription is subject to positive control of HrpG and HrpX [35]. Therefore, structural, regulatory, and secretory components of the T3SS are potential targets of sRNAs in *Xcc*.

It is unclear whether *Xoo* also deploys the functional mechanism of sRNAs targeting the T3SS due to paucity in related studies. *Xoo* is an important plant pathogenic bacterium, causing bacterial blight of rice, which is a major disease in China southern rice cultivation area and also in the southeast of Asia. The disease occurs mostly in rice leaves at the later stage of rice growth and is a severe threat to the high and stable yield of rice. Like in all plant pathogenic Gram-negative bacteria, *Xoo* uses the T3SS to secrete decades of effector proteins and translocate them into the cytosol of rice cells, where effectors execute their pathological roles [33, 36]. As a well-studied example, the *Xoo* strain PXO99^A^ confers virulence to the susceptible rice variety Nipponbare through the function of PthXo1, a transcription activator-like (TAL) protein, in manipulating rice transcriptome [37]. Evidently, an unappreciated functional relationship exists between certain sRNAs and effector secretion by the T3SS. However, this notion is not examined until now due to limited knowledge on sRNAs of plant pathogenic bacteria. To date, only eight sRNAs have been identified and shown to associate with various processes characterized by proteomics profiling analysis [29], but their pathological functions remain unclear. In conjecture, the genetic repertoire of *Xoo* is not likely to possess only eight sRNAs since hundreds of sRNAs exist in animal-pathogenic bacteria [3]. Clearly, it is a great challenge to determine the number and biological functions of sRNAs in *Xoo*.

This study is devised to determine the repertoire and virulence-associated members of *Xoo* sRNAs by the genomic sRNA-Seq technique performed with *Xoo* bacterial cultures under standard growth conditions in contrast to the medium that induces virulence factor production. High-throughput sequencing and bioinformatics analysis identify a total of 601 sRNAs in the bacterial genome and 12 out of them alter expression levels with virulence induction in contrast to the standard growth condition. Genetic analysis of mutagenesis and complementation suggests that two sRNAs, trans217 and trans3287, are essential for the bacterial virulence. We present evidence that trans217 and trans3287 act on the T3SS and effector secretion, providing the first case study of *Xoo* sRNAs targeting the effector secretion mechanism.

## Results

### Aggregate information on the genomic profile of sRNAs

**Fig. 1:**
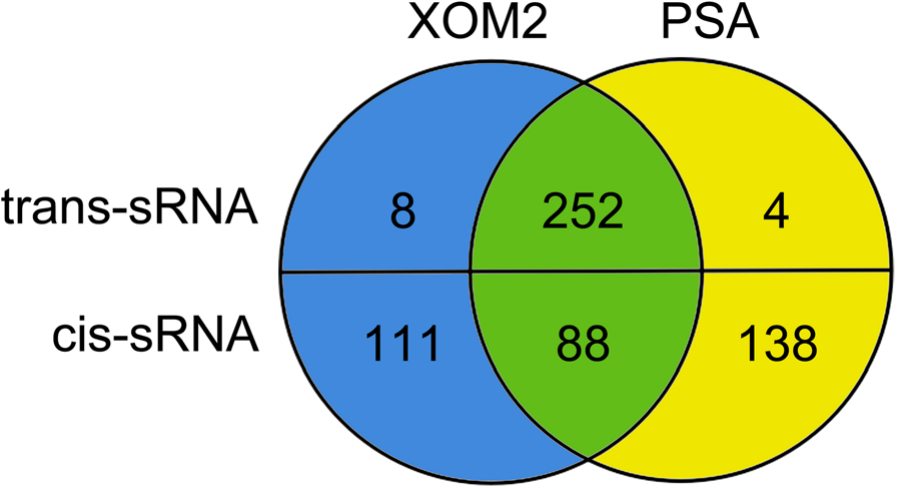
Venn diagram illustrating the sRNA composition in *Xanthomonas oryzae* pv. *oryzae* (*Xoo*) cultures on the medium PSA (polypeptone sucrose agar for normal growth) and the medium XOM2 (*X. oryzae* recipe 2 for virulence induction). The RNA-Seq identified 601 sRNAs in total. They were grouped according to categories, cis- or trans-sRNAs and media, PSA or XOM2

The genomic cDNA libraries of *Xoo* used in sRNA-Seq were prepared from cultures of the international standard strain PXO99^A^ grown on the standard growth medium polypeptone sucrose agar (PSA) or *X. oryzae* growth medium recipe 2 (XOM2), which induces gene expression related to the bacterial virulence [38]. The sRNA cDNA libraries were normalized to the size ranging from 50 to 500 nt and analyzed by a high-throughput sequencing technique to yield sRNA reads [39]. The number of sRNA reads from XOM2 was found to be less than that of PSA, suggesting that virulence induction impairs the expression of potential sRNAs in the bacterial genome. The number of sRNAs was determined as 601 in total, including 337 cis-sRNAs and 264 trans-sRNAs (Fig. 1; Table 1). Based on the different mechanisms by which cis- or trans-sRNAs function on targets [40], this sRNA classification information was used subsequently in the study to elucidate pathological functions of any of those *Xoo* sRNAs.

**Table 1:** Aggregate information on the genomic profile of *Xanthomonas oryzae* pv. *oryzae* sRNAs

	Bacterial cultures from^a^	
	PSA	XOM2
Total number of reads		
Abundant	14,287,830	12,748,596
Unique	7,143,915	6,374,298
Mapped to *Xoo* PXO99^A^ genome		
Total	12,415,866 (86.90%)	12,215,742 (95.82%)
Multiple mapped	943,376 (6.60%)	1,340,672 (10.52%)
Uniquely mapped	11,472,490 (80.30%)	10,875,070 (85.30%)
Number of candidate sRNA	601	
Total	482	459
Trans-	256	260
Cis-	226	199

^a^*PSA* a medium used for *Xoo* regular growth, *XOM2* a medium used to induce expression of *Xoo* genes associated with virulence

### Identification of virulence-associated sRNA candidates

**Fig. 2:**
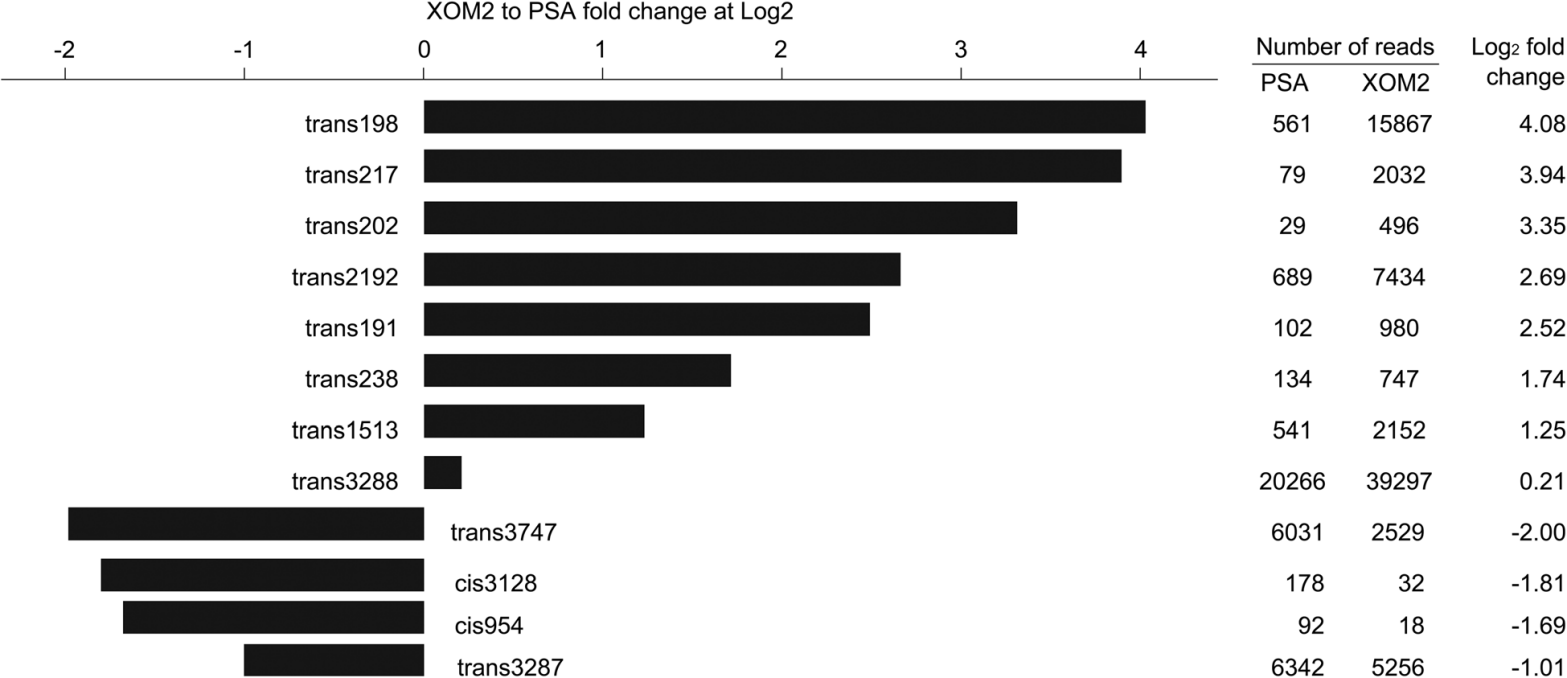
*Xoo* sRNAs differentially expressed under two culture conditions. Presented sRNAs were identified by high-throughput sRNA sequencing of RNA samples from *Xoo* cultures on PSA and XOM2 media, respectively. Fold change was calculated as Log2 ratio of sRNA transcript quantity between XOM2 and PSA cultures. The expression level of every sRNA was normalized to be the transcript per million (TPM)

In the genomic profiling of *Xoo* sRNAs from PSA and XOM2 cultures, expression levels of 10 sRNAs were found to alter between both cultures and quantified as XOM2-to-PSA fold changes in base-2 logarithms (Fig. 2; Additional file 1: Table S1). These sRNAs were subjected to deletion mutagenesis and virulence test. Meanwhile, trans3287 and trans3288 with high counts under both culture conditions (Additional file 1: Table S1) were also selected in the future studies to test whether or not they associate with virulence. The profiling values of XOM2-to-PSA fold changes (Fig. 2) were utilized to estimate degrees by which the tested sRNAs possibly associate with the bacterial virulence, which is induced by the XOM2 medium [38]. According to this criterion, trans198, trans217, and trans202, showing greater than 3-fold XOM2-to-PSA increases, were most positively relevant to virulence. Relatively less relevance to virulence was found in trans238, trans1513, and trans3288, which had XOM2-to-PSA fold changes of smaller than 2. The intermediate sRNAs were trans2192 and trans191, with fold increase values being 2.69 and 2.52, respectively. By contrast, four sRNAs were negatively associated with the bacterial virulence. In particular, the expression level of trans3747 was highly reduced by XOM2, showing the lowest XOM2-to-PSA fold decrease of 2 (Fig. 2).

**Fig. 3:**
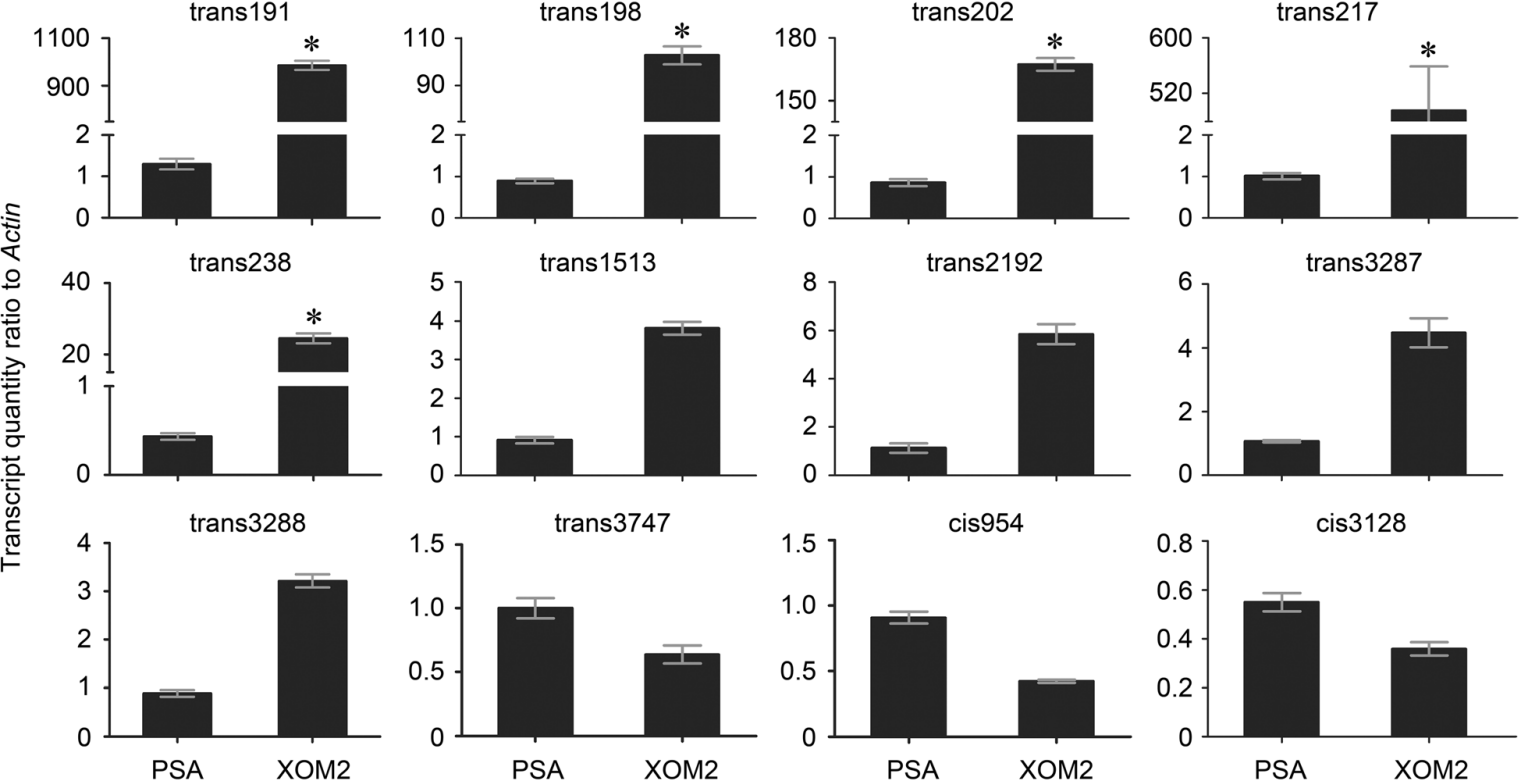
RT-qPCR (quantitative real-time reverse transcriptase polymerase chain reaction) analysis of selected sRNAs. Tested sRNAs were selected according to Fig. 1. RT-qPCR was carried out by using the constitutively expressed *16S rRNA* gene as a reference. Bar graphs represent mean values ± standard deviation (SD) error bars of data from 6 independent experiments (*n* = 6). Asterisks indicate significant differences between data from PSA and XOM2 cultures based on analysis of variation using Fisher’s least significant difference test (*P* < 0.01)

These results from the sRNA-Seq profiling were confirmed by RT-qPCR, namely real-time quantitative reverse transcriptase polymerase chain reaction (RT-qPCR). The relative level of sRNA gene expression was quantified as the ratio of sRNA to *16S rRNA*, a gene used as a reference owing to its characteristics of constitutive expression [41]. As shown in Fig. 3, RT-qPCR data indicated that relative amounts of trans191, trans198, trans202, trans217, and trans238 transcripts from XOM2 cultures were increased by dozen to thousand times compared to those from PSA cultures. Several fold increases or decreases were detected for the other 7 sRNAs (Fig. 3), in agreement with Log2 XOM2-to-PSA fold changes found in the genomic profile (Fig. 2).

Both the sRNA-Seq and RT-qPCR data suggests that trans191, trans198, trans202, and trans217, and trans238 are highly responsive to the culture condition that induces gene expression related to bacterial virulence. These sRNAs are regarded as candidates of virulence-associated sRNAs.

### Trans217 and trans3287 determine the bacterial virulence on rice

**Fig. 4:**
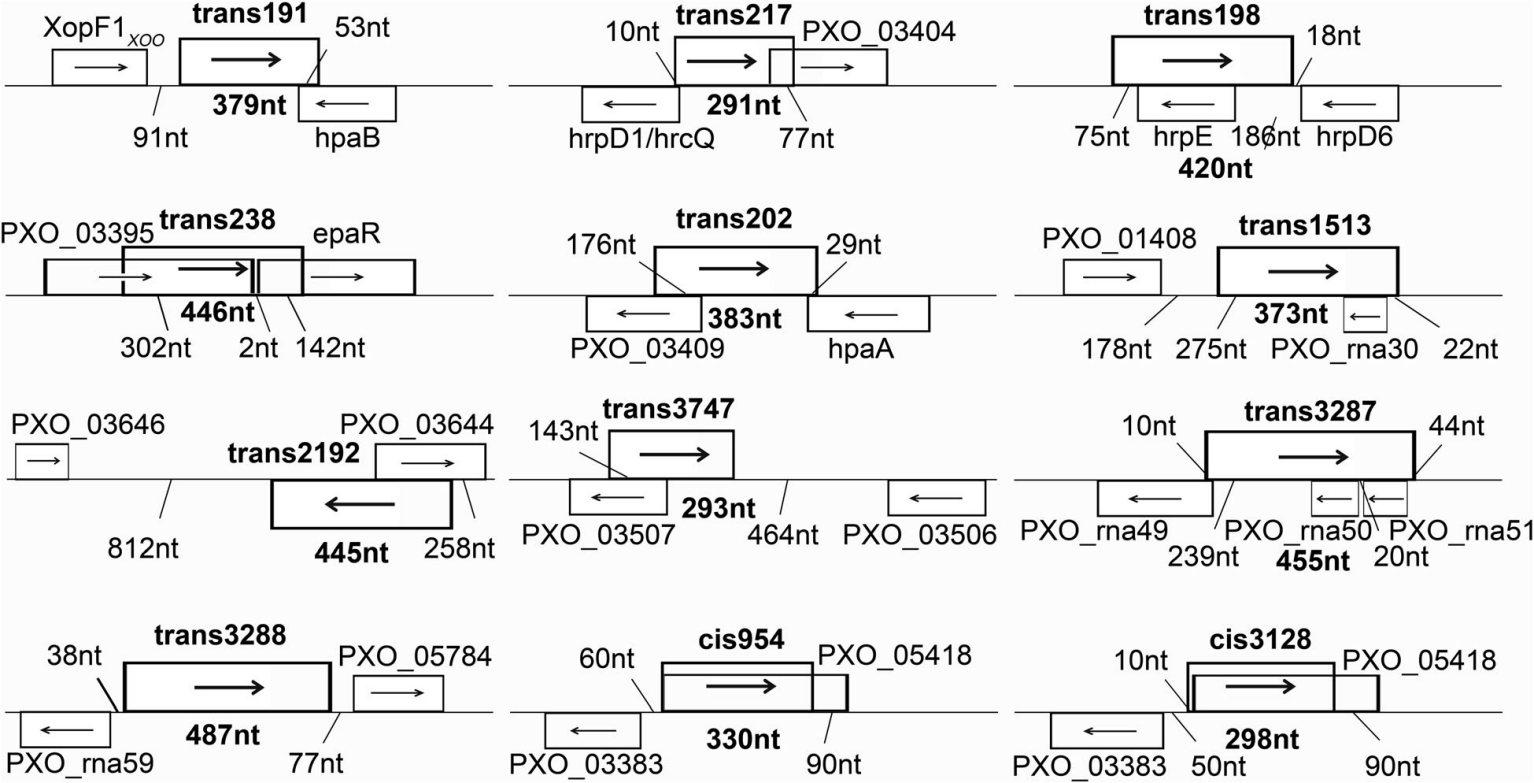
Schematic presentation of the genomic positions of selected sRNA genes. Gene positions were determined by NCBI blast against adjacent sequences of published *Xoo* genes

By NCBI blasting against adjacent sequences of published *Xoo* genes, length of the 12 sRNAs identified in this study varies from 291 to 487 nt and locate between or on known gene sequences at the bacterial chromosome (Fig. 4). This information was used to construct the bacterial sRNA-knockout mutants by dual-exchange of homologous arms.

**Fig. 5:**
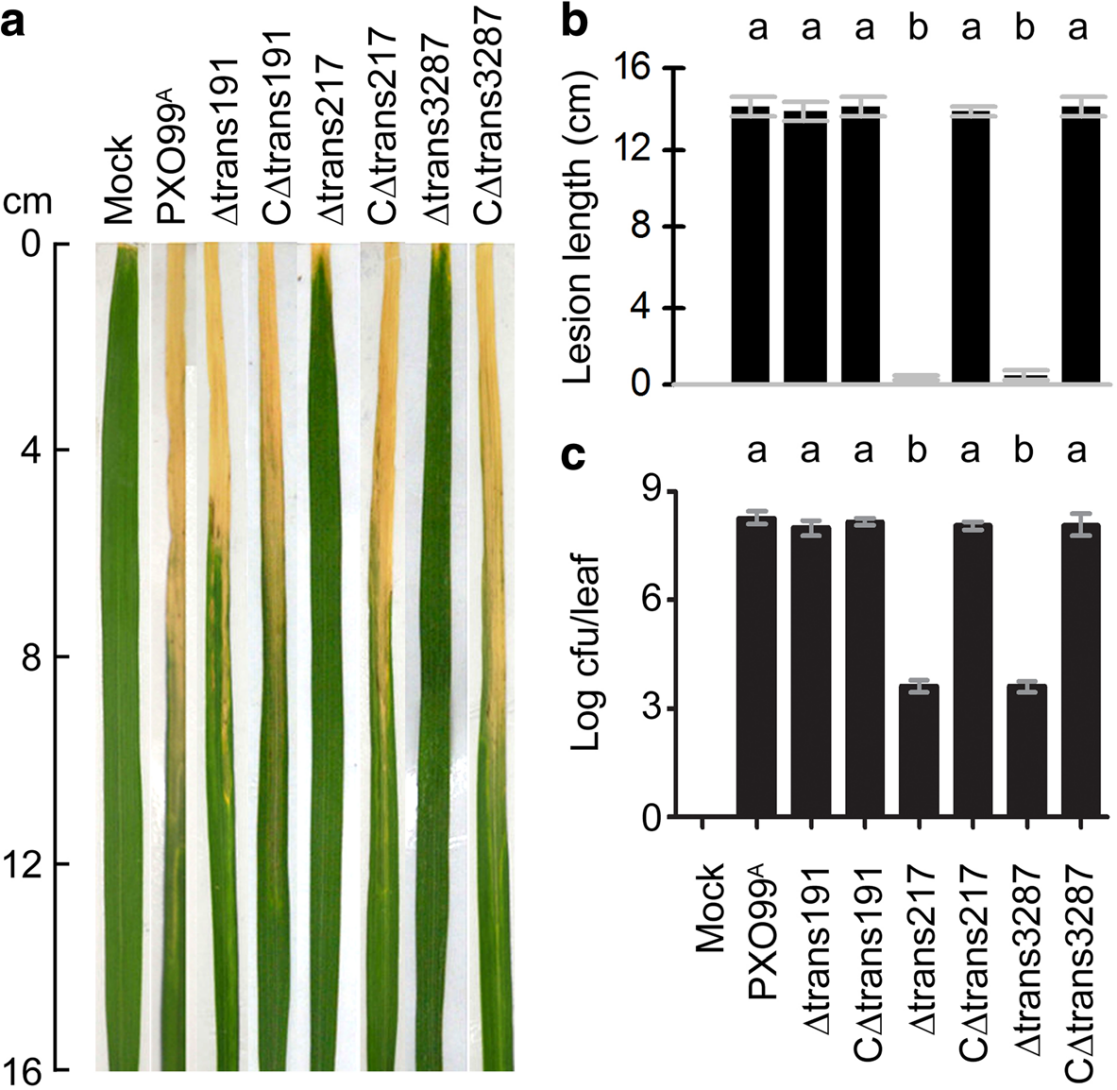
Virulence assessments of the wild-type (WT) strain PXO99^A^ and sRNA deletion mutants by inoculation and mock-inoculation experiments performed on the susceptible rice variety. **a** Bacterial blight symptoms on Nipponbare leaves photographed at day 14 after leaf-top-clipping inoculations. **b** Blight lesion length on leaves from (**a**). **c** Bacterial populations in Nipponbare leaves 3 days after leaf-center infiltrating inoculations. Data shown are means values ± SD bars [*n* = 10 leaves in (**b**); *n* = 3 experimental replicates in (**c**)]. Different letters in small case indicate significant differences by analysis of variation using Fisher’s least significant difference test (*P* < 0.01)

For use in analyzing whether any of the 12 virulent sRNA candidates is certainly involved in the bacterial virulence on the susceptible variety Nipponbare, every sRNA sequence was deleted from the PXO99^A^ genome by dual-exchange of homologous arms. Every sRNA deletion mutant was compared with the wild-type (WT) PXO99^A^ strain in virulence assessments by inoculation experiments. Great variations were found between the different sRNAs with respect to the effects of gene deletion on the bacterial virulence (Additional file 2: Figure S1). Virulence was shown as induction of the bacterial blight symptom (Fig. 5a), the disease severities (Fig. 5b), and *Xoo* populations propagated in tissues of inoculated leaves before the symptom development (Fig. 5c). Based on the genetic analysis of 12 sRNAs, only trans217 and trans3287 had relevance to the bacterial virulence, as evidenced by the virulence level reduction by gene deletion, shown as Δtrans217 and Δtrans3287. All the other 10 sRNAs did not show evident relevance to the bacterial virulence tested in comparative experiments.

**Fig. 6:**
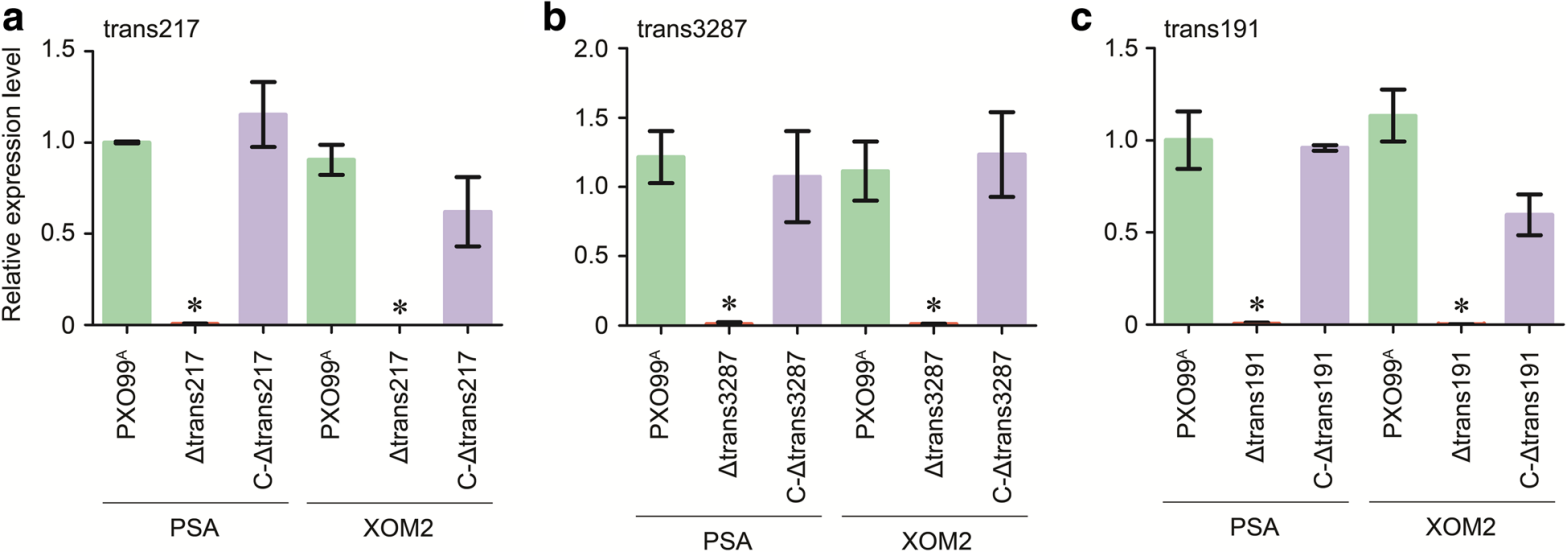
RT-qPCR analysis of sRNA gene expression in the different bacterial strains cultured on two media. Relative expression levels of (**a**) trans217, **b** trans3287, and (**c**) trans191 genes were given as ratios of sRNAs to *16S rRNA* transcript amounts. Data shown are mean values ± SD bars (*n* = 3). Asterisks indicate significant differences between the mutant and WT strain grown on the corresponding medium, based on analysis of variation using Fisher’s least significant difference test (*P* < 0.01)

**Fig. 7:**
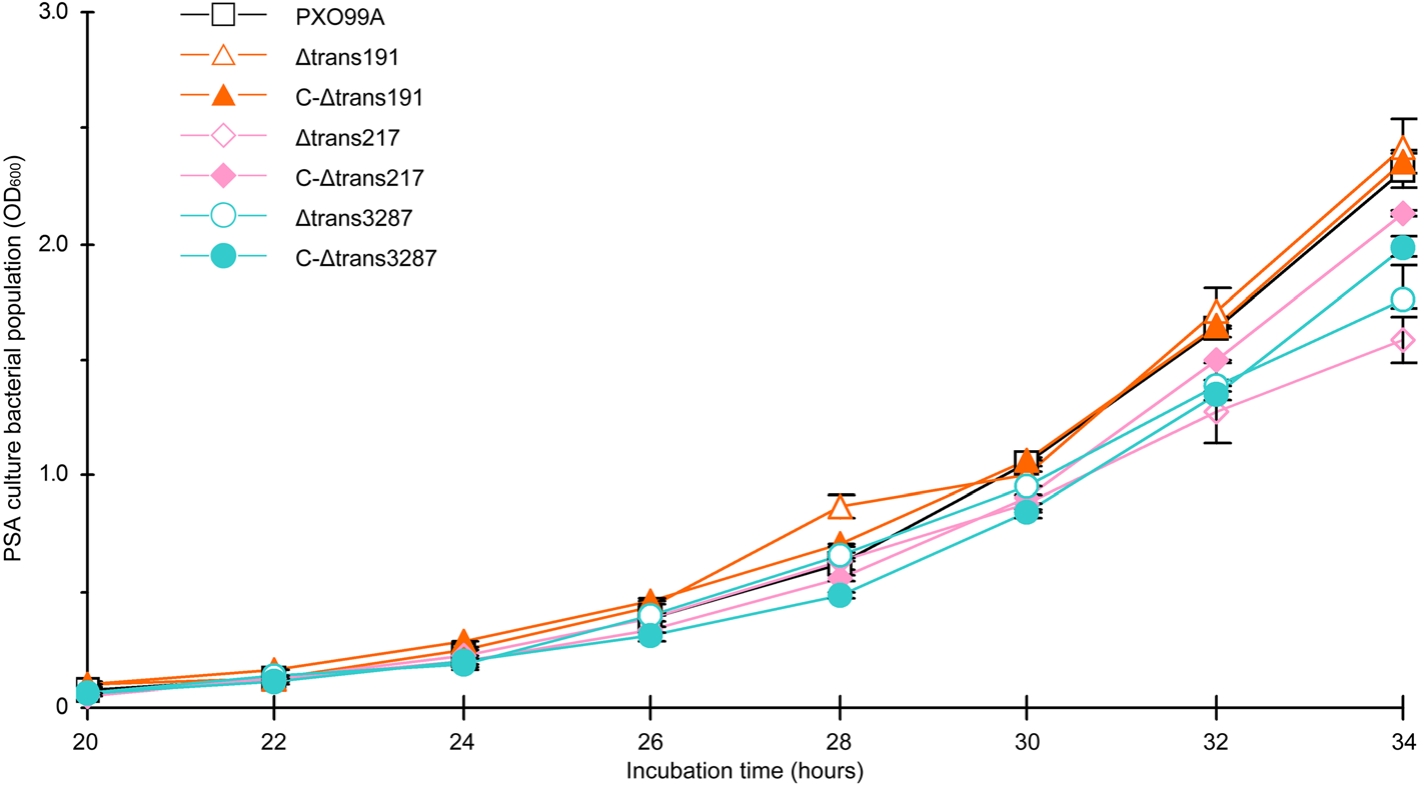
Bacterial growth curves. The values of OD_600_ represent for bacterial cells density. Data at each time point means values ± SD bars (*n* = 5)

The virulence compromise was attributed to the gene knockout (Additional file 2: Figure S1), which was confirmed by PCR analysis of the bacterial genomic DNA. Consistently, transcripts of both trans217 and trans3287 were not detected in RT-qPCR analysis of RNAs from cultures either from PSA or from XOM2 in corresponding mutant (Fig. 6a, b). Both PCR and RT-qPCR analyses also conform gene deletion of virulence-unrelated sRNAs, such as trans191 (Fig. 6c). Moreover, trans217 or trans3287 gene deletion did not affect bacterial multiplication on the growth medium (Fig. 7). These data offer additional evidence that regulation of the bacterial virulence is a predominant function of trans217 and trans3287 during infection of the susceptible rice variety.

This notion was corroborated by genetic complementation experiments. The original sequences of trans217 and trans3287 cloned from the WT PXO99^A^ genome was returned back into the genome of Δtrans217 and Δtrans3287, respectively. Resulting complement strains CΔtrans217 and CΔtrans3287 were restored to the WT not only in gene expression extents (Fig. 6), but also in virulence levels (Fig. 5a–c). In the parallel experiments, Δtrans191 was also complemented and tested similarly as for trans217 and trans3287 as trans191 is representative of virulence-unrelated *Xoo* sRNAs, with expression highly induced by XOM2 (Figs. 2 and 3) and without evident role in the bacterial virulence (Fig. 5a–c). The performances of trans191 in these experiments were constant, the virulence level of CΔtrans191 bacteria was similar to PXO99^A^. This was evidenced by equivalent severities of bacterial blight symptom (Fig. 5a), as well as equivalent quantities of blight lesion length (Fig. 5b) and bacterial population (Fig. 5c) in Nipponbare leaves irrespectively of inoculation with the different bacterial strains. These analyses strongly suggest that *Xoo* sRNAs showing high expression levels with the virulence induction are not necessarily related to the bacterial virulence but both trans217 and trans3287 definitely play a predominant role in the bacterial virulence.

### Trans217 and trans3287 function for the HR induction in tobacco

**Fig. 8:**
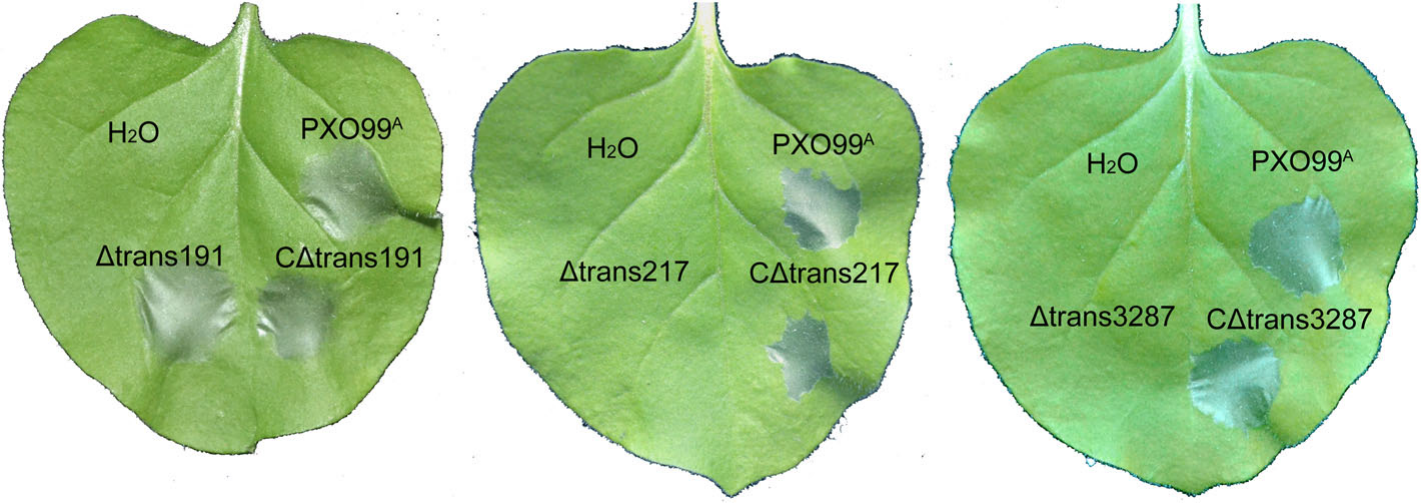
The hypersensitive response assays. Tobacco leaves are infiltrated with an aqueous bacterial suspension or water and photographed 2 days later

In agreement with the original definition about the hypersensitive response and pathogenicity (*hrp*) gene cluster present in plant-pathogenic Gram-negative bacteria [36], the presence of an *hrp* cluster enables the WT PXO99^A^ strain to induce the HR in tobacco *N. benthamiana* leaves [42]. However, not all *hrp* genes contribute to the HR induction [43]. Therefore, we investigated whether the HR induction involves trans217 and trans3287 by using the conventional method of infiltrating a bacterial suspension into the apoplastic space of tobacco leaves. We found that deleting trans217 or trans3287 nullified the bacterial capability of inducing the HR, whereas, genetic complementation of Δtrans217 and Δtrans3287 returned the HR induction (Fig. 8). Once again, the Δtrans191 or CΔtrans191 strain performed like the WT strain, eliciting a full development of the HR in tobacco leaves (Fig. 8). This result rules out the functional association of trans191 with the HR induction and suggests that both trans217 and trans3287 function in the way of certain *hrp* components to induce the HR.

### Trans217 and trans3287 are needed for *hrpG* and *hrpX* expression

**Fig. 9:**
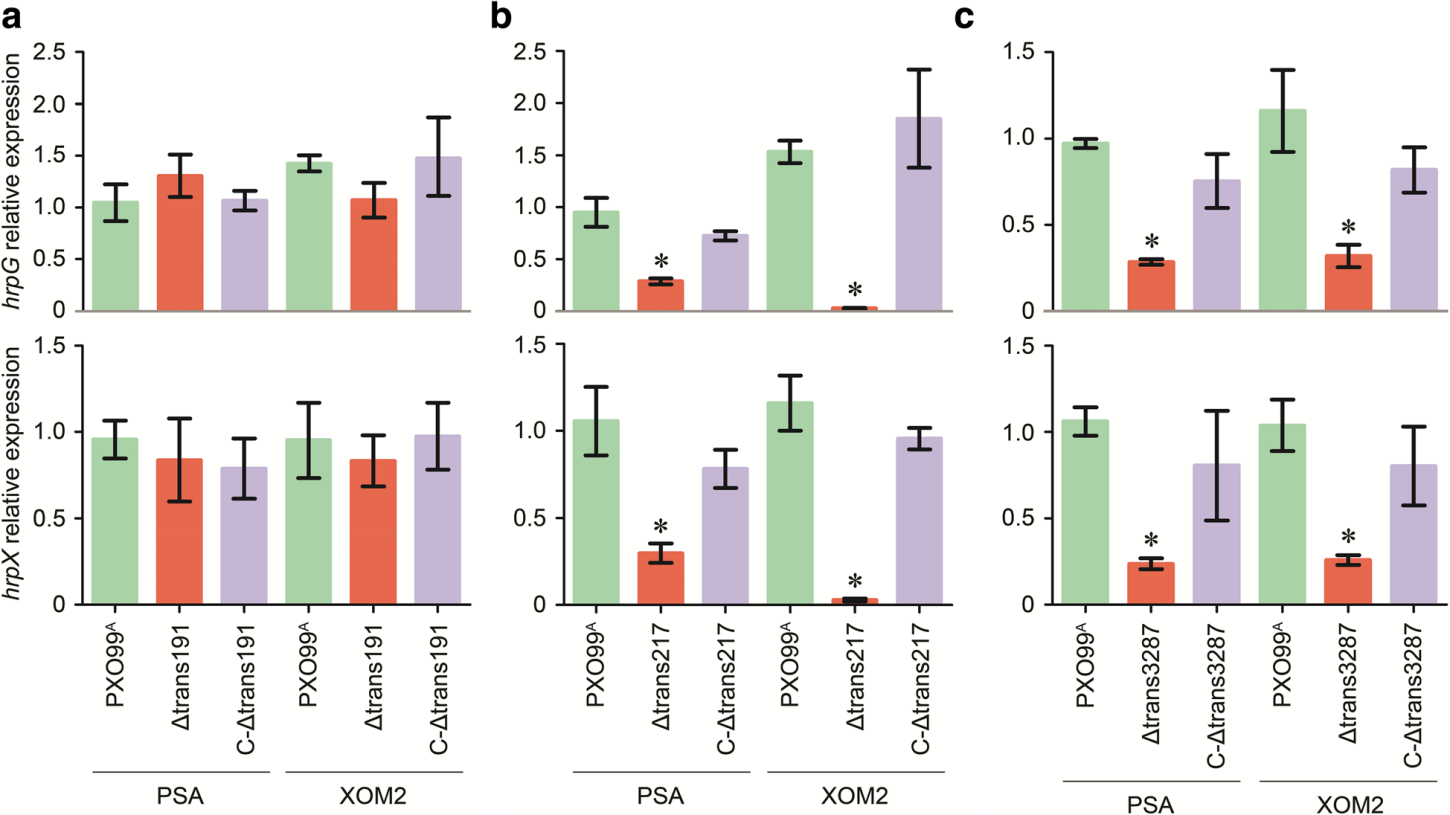
RT-qPCR analysis of the *hrp* genes expression in the different bacterial strains cultured on two media. Relative expression levels of *hrpG* and *hrpX* in bacterial strains related to (**a**) trans191, **b** trans217, and (**c**) trans3287 were given as ratios of the *hrp* to *16S rRNA* transcript amounts. Data shown are mean values ± SD bars (*n* = 3). Asterisks indicate significant differences between the mutant and WT strain grown on the corresponding medium, based on analysis of variation using Fisher’s least significant difference test (*P* < 0.01)

Once the *hrpG* and *hrpX* genes are expressed, their encoding products, regulatory proteins HrpG and HrpX, are essential for the subsequent expression of other *hrp* genes that encode structural and functional components of the T3SS machinery [32, 44]. To infer the functional relationship between the virulent sRNAs and *hrp* genes, we carried out RT-qPCR analysis to compare expression levels of *hrpG* and *hrpX* genes in PSA and XOM2 cultures of *Xoo* strains with and without trans191, trans217, and trans3287, respectively. Based on the ratios of sRNA to *16S rRNA* transcript quantities, both *hrpG* and *hrpX* were expressed to equivalent degrees in the WT, Δtrans191, and CΔtran191 strains irrespectively of culture in PSA or XOM2 (Fig. 9a). However, significant decreases of *hrpG* and *hrpX* expression levels were caused by Δtrans217 or Δtrans3287 compared to the WT or complementary strains (Fig. 9b, c). Evidently, trans217 and trans3287 take part in *hrpG* and *hrpX* expression but trans191 does not.

### Trans217 and trans3287 participate in PthXo1 secretion

**Fig. 10:**
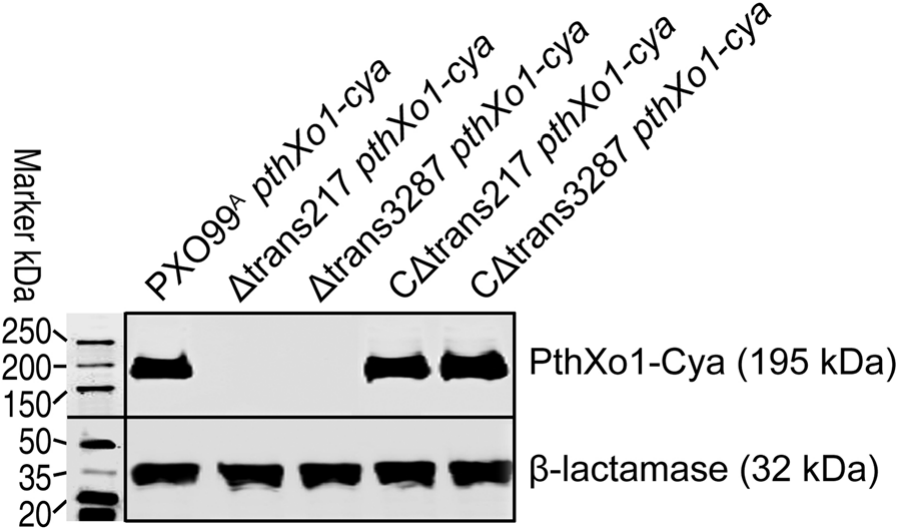
PthXo1-Cya fusion protein secretion assay. Bacterial cultures of the strains shown on top of gel were used to isolate secreted proteins. Protein blot was hybridized with the Cya antibody. Blot of intracellular proteins prepared from the same bacteria was probed by the antibody against the intracellular protein β-lactamase used to indicate protein production in the bacterial, an intracellular protein marker

Since HrpG and HrpX regulate the expression of other *hrp* genes that encode T3SS accessory and secreted proteins [45], trans217- and trans3287-dependent expression of *hrpG* and *hrpX* gene expression may have subsequent effect on T3SS effector secretion. To test the hypothesis, we analyzed the effects of both sRNAs on the PthXo1 secretion, which was monitored by using calmodulin-dependent adenylate cyclase (Cya), a eukaryotic cytoplasmic import marker [46]. As shown in Fig. 10, PthXo1-Cya was highly secreted out of cells in the *Xoo* WT strain, whereas, the fusion protein secretion was totally cancelled in Δtrans217 and Δtrans3287 mutants, which were created by deletion of trans217 and trans3287 accordingly from the bacterial genome. By contrast, when the Δtrans217 and Δtrans3287 mutants were complemented, PthXo1 secretion out of bacterial cells of complementation CΔtrans217 and CΔtrans3287 strains was brought back to the WT level (Fig. 10). Clearly, trans217 and trans3287 are critical to PthXo1 secretion, providing the first case that connects bacterial sRNAs with effector secretion.

## Discussion

By the genomic sRNA-Seq profiling and experimental genetic analysis, one of the main findings in this study is the importance of trans217 and trans3287 to the virulence of *Xoo* strain PXO99^A^ on the susceptible rice variety Nipponbare. We show that both sRNAs function as key components of the bacterial virulence. The nullification and restoration of the bacterial virulence correspondingly by deleting trans217 or trans3287 and by the genetic complementation suggest that trans217 and trans3287 are virulent sRNAs present in the *Xoo* genome. This information adds useful insight into the current understanding of *Xoo* sRNAs, which are paid attention only in recent years [47]. To date, merely a few sRNAs have been characterized in the *Xoo* genome while none of them was definitely related to the bacterial pathogenicity or virulence [29].

To infer the molecular mechanism that underpins the pathological function of trans217 and trans3287, we have correlated the virulent role of both sRNAs with their positive effects on the expression of *hrpG* and *hrpX* genes. Both genes reduce expression quantities in the bacterial Δtrans217 and Δtrans3287 mutants but are highly expressed in the genetically complemented bacteria as in the WT strain. This result sheds a light into exploring molecular targets of trans217 and trans3287 while we do not have evidence at present to demonstrate how the sRNAs affects *hrpG* or *hrpX* expression. There is no homology present in trans217 and trans3287 sequences based on Blast Search. Analysis with TargetRNA2 (http://cs.wellesley.edu/~btjaden/TargetRNA2/) suggests that *hrpG* and *hrpX* are not targets of trans217 and trans3287. Instead, several predicted targets of trans217 and trans3287 are related to the T3SS apparatus formation or biochemical reactions. Thus, the effects of both sRNAs on *hrpG* and *hrpX* are indirect and involved mechanisms remain to be studied.

In the protein form, HrpG and HrpX are essential regulators of T3SS and execute the regulatory function at the transcription level [48, 49]. HrpG regulates the expression of *hrpX* gene while subsequently produced HrpX protein acts in turn to regulate the expression of other *hrp* genes, which encode T3SS accessory proteins like Hpa1 [50, 51] and effector proteins like PthXo1 secreted through the T3SS pathway [37]. It was recently found that the *Xcv* sRNAs sX13 took part in the expression of *hrpX* in correlation with the virulent function of sX13 [32]. Thus, targeting T3SS regulators is likely to be a common mechanism conserved in the virulence function of certain sRNAs from plant-pathogenic bacteria of the *Xanthomonas* genus. It is apparent that trans217 and trans3287 differ from sX13 in terms of direct molecular targets. Irrespective of the direct gene targets, however, the effect of sRNAs on T3SS components establishes a mechanistic connection between the sRNAs and bacterial virulence performance though particular effectors [52].

In agreement with the assumed functional connection, we demonstrate that trans217 and trans3287 are required for PthXo1 secretion from the bacterial cells outward to the culture environment. This finding indicates that targeting effector secretion may represent one of the molecular mechanisms by which bacterial sRNAs perform their virulent functions. An alternative mechanism has been characterized to be the suppression of immune responses in plants [47]. Because PthXo1 determines the virulence of *Xoo* strain PXO99A on the susceptible rice variety Nipponbare [37], the apparent role of sRNAs in the control of PthXo1 secretion is obviously indirect but crucial for the effector to be translocated into the plant cell [33, 37]. Translocation from bacterial cells into the cytosol of plant cells is a key step for effectors to fulfil their pathological roles [33, 53, 54]. Effector translocation indispensably needs direct mediation by type III translocators, such as Hpa1 [33]. Like HrpG and HrpX, type III translocators also belong to T3SS components but are closer than both Hrp proteins in the spatial and temporal patterns of functions from secretion to translocation of effectors [33]. Therefore, sRNAs regulate bacterial virulence by complicated functional networks, which remain to be studied in the future. In the future, it is especially necessary to elucidate whether trans217 and trans3287 function during the *Xoo*-rice interaction process or during rice infection by the pathogen.

## Conclusions

Based on the bacterial performance in the absence and presence of trans217 and trans3287, trans217 and trans3287 serve as pathogenicity-associated sRNAs essential for the bacterial virulence on the susceptible rice variety and for the HR elicitation in the nonhost plant. Gene expression and protein secretion data offer the molecular evidence suggesting that both virulent sRNAs regulate the bacterial virulence by targeting the type III secretion system.

## Methods

### Plant growth and bacteria cultures

Seeds of rice *Oryza sativa L. japonica* variety Nipponbare were sown in pots filled with a mixture of sand and potting soil (1:1 *v*/v) and seedlings were grown in a plant growth chamber under 25–26 °C, 85% humidity, and a 14-h light cycle at 250 μE/m^2^/s. Seeds of tobacco *Nicotiana benthamiana* were sown in the soil mixture as for rice and plants were grown in a different plant growth chamber with controlled environment as for rice growth. The WT *Xoo* strain PXO99^A^ and different sRNA-related strains (Additional file 3: Table S2) are maintained in this lab. Bacteria were cultured in liquid nutrient broth (NB) medium in a 28 °C shaking incubator for preparation of inoculum while bacteria isolated from plant leaves were incubated in nutrient broth agar (NB) medium. Alternatively, bacteria were cultured separately on PSA and XOM2 media [38] for use in sRNA sequencing.

### RNA isolation and quality optimization

Nursery bacterial PSA or XOM2 culture suspension with a desirable density (OD_600_ ≈ 0.5) was supplied to fresh medium and incubated in a 28 °C shaker for 6 h. Bacterial cells were collected by centrifuge and employed to isolate total RNA using the trizol reagent kit (Invitrogen) as per the manufacturer’s protocol. In order to eliminate possible variations between bacterial cultures, 2 biological repetitions were performed. In each repetition, RNA was isolated from a mixture of five PSA or XOM2 cultures and purity was assessed using the Nanodrop Spectrophotometer ND-1000 (Nanodrop Technologies). Each RNA sample had an A260:A280 ratio greater than 1.8 and A260:A230 ratio higher than 2.0. RNA integrity was evaluated using the Agilent 2200 TapeStation (Agilent) and each sample was quarantined to have the RIN (RNA Integrity Number) greater than 7.0. Then, RNAs were ligated with 3’-RNA adapter, followed by 3′-adapter ligation. Subsequently, the adapter-ligated RNAs were subjected to RT-PCR and amplified with a low-cycle program (10 cycles). To obtain 50–500 nt RNA, the PCR products were fractioned by PAGE according to instructions of NEBNext® Multiplex Small RNA Library Prep Set for Illumina® (Illumina). Finally, the purified library products were evaluated using the Agilent 2200 TapeStation and diluted to 10 pM for cluster generation in situ on the HiSeq2500 single-end flow cell, followed by sequencing (1 × 50 bp) with the HiSeq 2500 facility.

### *Xoo* sRNA sequencing and sRNA identification

The PSA or XOM2-derived cDNA libraries were amplified by PCR and products were sequenced with the IlluminaHiSeq™2500 sequencer in a commercial company. Image data output from the sequencing device were converted into raw reads and stored in the FASTQ format. Clean reads were obtained after removing reads that contained adaptor sequences, reads containing more than 10% of unknown bases, and reads in which more than half of the quality values of the bases were less than 5. Sequences of the clear reads were mapped to the public PXO99^A^ genome (NC_010717.2) and the overlap reads were selected to splice sRNA fragments if the coverage depth at each base was higher than 50X. This also yield the information on sRNA sequence, length and orientation. These sRNA fragments were named as sRNA1, sRNA2, and sequential codes for more RNAs [39]. All sRNAs from the different samples (PSA and XOM2 cultures) were collectively analyzed to identify candidates of trans-sRNAs and cis-sRNAs according to their origin from the PXO99^A^ genome. A sRNA shorter than 500 nt and transcribed from the opposite strand of an mRNA or another sRNA was identified as cis-sRNAs while a sRNA shorter than 500 nt and transcribed from the intergenic region was identified as a trans-sRNA [39, 55].

### Verification of sRNAs differentially expressed in PSA and XOM2 cultures

Expression levels of sRNAs from both cultures quantified as average transcript levels as previously described [39]. Log 2 fold change ratios of XOM2 to PSA transcript quantities were calculated by the edgeR method [56] and assessed for significant differences at *P* < 0.01 when the ratio was greater than 1. This type of sRNAs were analyzed by RT-qPCR to confirm expression levels in the Seq data, using specific primers (Additional file 4: Table S3). In every RT-qPCR protocol, the 25 μl reaction mixture was composed of 1 μl first-strand cDNA diluted 1:10, 2.5 μM primer, and 1 × SYBR Premix Ex Taq (TaKaRa). All reactions were performed in triplicate with null-template controls in which cDNA was absent. The constitutively expressed *16S rRNA* gene was used as a reference, and relative expression level of an sRNA was quantified as the ratio of sRNA to *16S rRNA* transcript amounts.

### The sRNA-directed mutation and complementation

To generate sRNA deletion PXO99^A^ mutants, flanking sequences of up- and downstream of *sRNA* were amplified by PCR from PXO99^A^ using primers (Additional file 4: Table S3). PCR products were digested with respective restriction enzyme and ligated into the suicide vector pK18mabSacB (Additional file 3: Table S2). The recombinant vectors were transferred into PXO99^A^ competent cells by electroporation. Transformed bacterial cells were incubated in sugar-absent NA plates containing 100 μg/mL kanamycin under 28 °C for 3 days and then single clones were verified by PCR. The suspension of positive clones was smeared on plates of NA medium containing 10% sucrose, incubated for 3 days, and subjected to PCR analysis to identify sRNA deletion mutants. Complemented strains were created by sRNA PCR fragment ligating into the expressive vector pHM1 via appropriate restriction enzyme (Additional file 4: Table S3). Every recombinant vector was transferred into the competent cells of the corresponding sRNA deletion mutant. Transformants were identified similarly as for mutants.

### Virulence assessments

In plant inoculation assay, the leaves of two-month old rice were clipped using sterile scissors which were dipped in the bacterial cultures (OD_600_ ≈ 0.5) at a distance of about 2 cm from the leaves edge. Each strain was inoculated in five plants and three leaves of each plant were tested. Lesion length was scored 2 weeks after inoculation and the average was calculated. To determine bacterial population in leaf tissues, leaves were segmented, sterilized with 75% (*v*/v) ethanol, and homogenized in sterile water; bacteria were recovered from the resulting homogenates by culturing on NA medium. Bacterial number in leaves was gives as colony formation unit (cfu). At ultimate, virulence levels of different *Xoo* strains were evaluated by bacterial blight severity (lesion length) and bacterial populations in leaf tissues.

### Bacterial growth assessment

Overnight *Xoo* strains PSA cultures (OD_600_ ≈ 1.0, 10 uL) were corresponding pipetted to a new bottle of 20 mL medium, cultured in a shaking incubator under 28 °C. Bacterial populations were measured every 2 h during 20–34 h.

### The HR induction

The activated bacterial cultures were pipetted to NB medium until the optical density (OD_600_ ≈ 0.5). Bacterial suspensions of the different strains were separately infiltrated by using needleless syringes into intercellular spaces of at the 5th and 6th tobacco leaves. Water was applied similarly in control. Two days later, infiltrated leaves were photographed.

### PthXo1 secretion assay

The recombinant PXO99^A^ sRNA *pthXo1-cya* strains were grown under 28 °C in liquid NB with 50 μg/mL spectinomycin to logarithmic phase. Bacterial cells were harvested by centrifugation. The precipitated bacterial pellet was washed twice with sterile water and resuspended in 100 mL XOM2 liquid media [38] with 50 μg/mL spectinomycin to OD_600_ = 0.6 and cultured in a 28 °C shaker at 220 *rpm* for 16 h. XOM2 cultures were then separated into cell pellet and supernatant fractions by centrifugation. The proteins in the two fractions were extracted by sonication and by precipitation with 12.5% trichloroacetic acid, respectively [57]. Proteins were separated by 6% SDS-PAGE and transferred to Immobilon-P membranes (Millipore) for immunoblotting analyses using a Cya antibody (Santa Cruz) or β-lactamase antibody (Abcam). The ampicillin resistance protein β-lactamase protein is encoded by the pHM1*-cya* vector, remains cell-bound unless non-specific cell leakage occurred, and was used as a control for nonspecific cell lysis. Protein blots were incubated with the specific antibody and hybridized to horseradish peroxidase-conjugated goat antimouse immunoglobulin G from the BeyoECL Plus kit (Beyotime).

### Statistical analysis

All experiments were conducted at least three times with similar results. Quantitative data were analyzed by using the commercial IBM SPSS19.0 software package [58]. Homogeneity-of-variance in data was determined by using the Levene test, and the formal distribution pattern of the data was confirmed by using the Kolmogorov-Smirnov test and P-P plots. Data were subjected to ANOVA, along with Fisher’s least significant difference test and Tukey-Kramer’s test, respectively. Significance was tested for differences in pair or multiple comparisons of different bacterial strains.

## Additional files

Additional file 1: **Table S1.** Information on 12 sRNA selected from the *Xanthomonas oryzae* pv. *oryzae* sRNA-Seq profiling. (DOCX 14 kb)

Additional file 2: **Figure S1.** Bacterial blight severities of the susceptible rice variety Nipponbare inoculated with sRNA-related strains. (DOCX 80 kb)

Additional file 3: **Table S2.** Strains and plasmids used and created in this study. (DOCX 15 kb)

Additional file 4: **Table S3.** Information on genes tested and primers used in this study. (DOCX 17 kb)

## Abbreviations

HR: Hypersensitive response
RT-PCR: Reverse transcriptase-polymerase chain reaction
RT-qPCR: Quantitative real-time RT-PCR
sRNAs: Small RNA
TAL: Transcription activator-like
*Xoo*: *Xanthomonas oryzae* pv. *oryzae*
