# Supplementary material for: Two virulent sRNAs identified by genomic sequencing target the type III secretion system in rice bacterial blight pathogen
Source: BMC Plant Biol. 2018 Oct 16;18:237. doi: 10.1186/s12870-018-1470-7 (PMC6192180; doi:10.1186/s12870-018-1470-7)
Supplement: Supplementary file 1 — Table S1. Information on 12 sRNA selected from the Xanthomonas oryzae pv. oryzae sRNA-Seq profiling. (DOCX 14 kb) [file 12870_2018_1470_MOESM1_ESM.docx]

**Supplemental Data**

**Additional file 1: Table S1.** Information on 12 sRNA selected from *Xanthomonas oryzae* pv. *oryzae* sRNA-Seq profiling.

| sRNA | start | end | PSA count | XOM2 count | PSA  expression | XOM2  expression | Log2 fold change | *P*_value |
| --- | --- | --- | --- | --- | --- | --- | --- | --- |
| trans198 | 66397 | 66817 | 561 | 15867 | 19.33854198 | 326.9925655 | 4.079706902 | 1.75E-08 |
| trans217 | 70552 | 70843 | 79 | 2032 | 3.926333631 | 60.37623187 | 3.942726047 | 5.33E-06 |
| trans202 | 67668 | 68051 | 29 | 496 | 1.095997930 | 11.20664475 | 3.354037424 | 0.014145884 |
| trans2192 | 4314541 | 4314986 | 689 | 7434 | 22.41957231 | 144.6148365 | 2.689384909 | 5.01E-05 |
| trans191 | 65622 | 66001 | 102 | 980 | 3.895467051 | 22.37523637 | 2.522034706 | 0.003832761 |
| trans238 | 79136 | 79582 | 134 | 747 | 4.350510642 | 14.49900606 | 1.736697351 | 0.029721761 |
| trans1513 | 3493991 | 3494364 | 541 | 2152 | 20.99271564 | 49.92244159 | 1.249799688 | 0.032496390 |
| trans3288 | 5088375 | 5088862 | 20266 | 39297 | 602.6861017 | 698.6581468 | 0.2131799223 | 0.306551346 |
| trans3747 | 5217667 | 5217960 | 6031 | 2529 | 297.7041976 | 74.63227007 | -1.996008090 | 0.027687831 |
| cis3128 | 5020680 | 5020978 | 178 | 32 | 472.4743855 | 134.7901906 | -1.809520610 | 0.001725982 |
| cis954 | 848808 | 849138 | 92 | 18 | 220.5917614 | 68.48950208 | -1.687424134 | 0.003774409 |
| trans3287 | 5086294 | 5086749 | 6342 | 5256 | 201.8386604 | 100.0036074 | -1.013150493 | 0.431326993 |
