# Supplementary material for: Two virulent sRNAs identified by genomic sequencing target the type III secretion system in rice bacterial blight pathogen
Source: BMC Plant Biol. 2018 Oct 16;18:237. doi: 10.1186/s12870-018-1470-7 (PMC6192180; doi:10.1186/s12870-018-1470-7)
Supplement: Supplementary file 2 — Figure S1. Bacterial blight severities of the susceptible rice variety Nipponbare inoculated with sRNA-related strains. (DOCX 80 kb) [file 12870_2018_1470_MOESM2_ESM.docx]

**Supplemental Data**

**
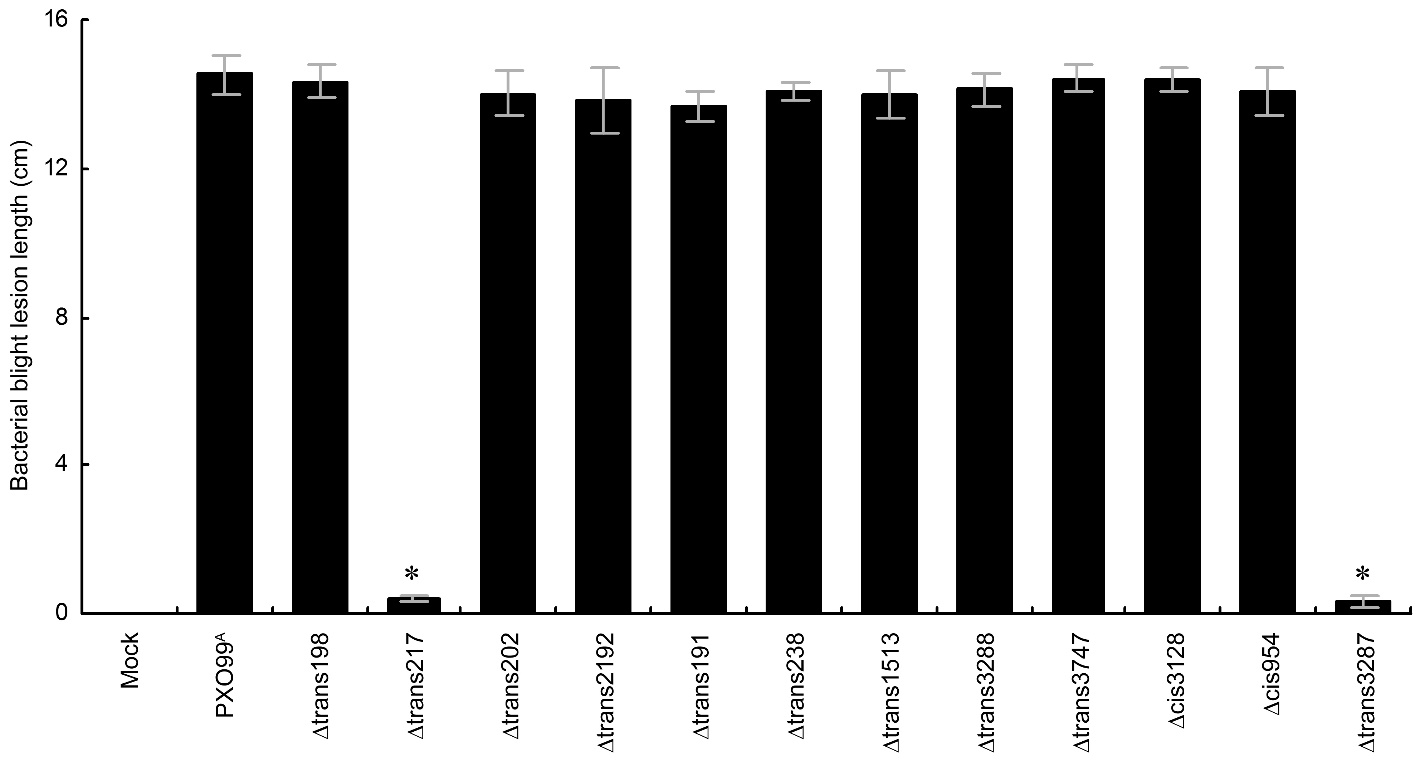
**

**Additional file 2: Figure S1.** Bacterial blight severities of the susceptible rice variety Nipponbare inoculated with sRNA-related strains. Leaves of two-month old rice plant were clipped using sterile scissors which had been dipped in the bacterial cultures (OD_600_ ≈ 0.5) at a distance of about 2 cm from the leaves edge. Each strain was inoculated in 5 plants and 10 leaves of each plant were tested. Lesion length was scored two weeks after inoculation and the average was calculated. Data shown are means ± standard deviation bars of 3 independent experiments, each containing 5 plants and 10 leaves. Asterisks indicate significant differences in multiple comparison of the data (*P* < 0.01).
