# Supplementary material for: Two virulent sRNAs identified by genomic sequencing target the type III secretion system in rice bacterial blight pathogen
Source: BMC Plant Biol. 2018 Oct 16;18:237. doi: 10.1186/s12870-018-1470-7 (PMC6192180; doi:10.1186/s12870-018-1470-7)
Supplement: Supplementary file 3 — Table S2. Strains and plasmids used and created in this study. (DOCX 15 kb) [file 12870_2018_1470_MOESM3_ESM.docx]

**Supplemental Data**

**Additional file 3: Table S2.** Strains and plasmids used and created in this study.

| Strains or Plasmids | Relevant characteristics | Source |
| --- | --- | --- |
| Strains |  |  |
| *Escherichia coli* DH5α | F^–^ 80d*lacZ* M15(*lacZYA-argF*) U169 *endA1 deo*R *rec*A1 *hsd*R17(r_K_^–^ m_K_^+^) *pho*A *sup*E44 λ^–^ *thi-l gyr*A96 *rel*A1 | This lab |
| *Xanthomonas oryzae* pv. *oryzae* PXO99^A^ | Philippine race 6; azacytidine resistant clone of PXO99, virulent to rice cultivars Nipponbare and IRBB10 | This lab |
| Δtran191 | PXO99^A^ *trans191* unmarked mutant | This study |
| Δtran217 | PXO99^A^ *trans217* unmarked mutant | This study |
| Δtran3287 | PXO99^A^ *trans3287* unmarked mutant | This study |
| Δtrans217/pthXo1-cya | PXO99^A^ *trans217* mutant transformed with pHM1-*pthXo1-cya* | This study |
| Δtrans3287/pthXo1-cya | PXO99^A^ *trans3287* mutant transformed with pHM1-*pthXo1-cya* | This study |
| CΔtran191 | PXO99^A^ *trans191* mutant complemented with pHM1-*trans191* | This study |
| CΔtran217 | PXO99^A^ *trans217* mutant complemented with pHM1-*trans217* | This study |
| CΔtran3287 | PXO99^A^ *trans3287* mutant complemented with pHM1-*trans3287* | This study |
| CΔtrans217/pthXo1-cya | PXO99^A^ *trans217* mutant complemented with pHM1*-pthXo1-cya* | This study |
| CΔtrans3287/pthXo1-cya | PXO99^A^ *trans217* mutant complemented with pHM1*-pthXo1-cya* | This study |
| Plasmids |  |  |
| pK18*sacB* | Suicide vector derivative from pK18mobGII, *sacB^+^*, Km^R^ | This lab |
| pHM1 | Broad-host range vector with pUC19 polylinker, Sp^R^ | This lab |
