# Supplementary material for: Two virulent sRNAs identified by genomic sequencing target the type III secretion system in rice bacterial blight pathogen
Source: BMC Plant Biol. 2018 Oct 16;18:237. doi: 10.1186/s12870-018-1470-7 (PMC6192180; doi:10.1186/s12870-018-1470-7)
Supplement: Supplementary file 4 — Table S3. Information on genes tested and primers used in this study. (DOCX 17 kb) [file 12870_2018_1470_MOESM4_ESM.docx]

**Supplemental Data**

**Additional file 4: Table S3.** Information on genes tested and primers used in this study

| Gene | Primers / subjects |
| --- | --- |
| *trans191* | Upstream homologous arm:  F: 5’-CGCGGATCCCAACGAACGTGCCGTGCTT-3’ (*Bam*H I),  R: 5’-CGGAATTCACAGACGCGACGACAGTGAC-3’(*EcoR I*),  Downstream homologous arm:  F: 5’-CGGAATTCGATTGATGCTGCGGCAAGATG-3’(*EcoR* I),  R: 5’-AACTGCAGCGACAACGCTAAGCAGCTCG-3’ (*Xba* I) / deletion |
|  | F: 5’-CCCAAGCTTCATTGCTTAAAGAGATCCGC-3’ (*Hind* III),  R: 5’-GGGGTACCGACGAGATGTTCGAAGGAAT-3’ (*Kpn* I) / complemention |
|  | F: 5’-CTGGCAGCTTAGAGCGGCGAA-3’  R: 5’-GACGAGATGTTCGAAGGAATCGC-3’ / RT-qPCR |
| *trans217* | Upstream homologous arm:  F: 5’-CGCGGATCCCGTGCAATGCAGCAAGAC-3’ (*BamH* I),  R: 5’-ATCGCATGCCTCACCCAATGATGCGG-3’(*Sph* I),  Downstream homologous arm:  F: 5’-ATCGCATGCCGCTGCCTTCCACACCAA-3’(*Sph* I),  R: 5’-AACTGCAGAGCGTTCCAGCTTCCTGC-3’ (*Pst* I) / deletion |
|  | F: 5’-CCCAAGCTTCGCAATTGCATCGAGATC-3’ (*Hind* III),  R: 5’-GGGGTACCCTGCACTGACGTCAACACTG-3’ (*Kpn* I) / complemention |
|  | F: 5’-CCGGTATCCACCTGCAGGC-3’  R: 5’-CACTGACGTCAACACTGAGTAGCAC-3’ / RT-qPCR |
| *trans3287* | Upstream homologous arm:  F: 5’-CGCGGATCCTTTTCGCCTTTCCCTCAC-3’ (*BamH* I),  R: 5’-ATCGCATGCAAGCGCACACGGTGGAT-3’(*Sph* I),  Downstream homologous arm:  F: 5’-ATCGCATGCATGCAGGTAATTTGTGAGGAC-3’(*Sph* I),  R: 5’-AACTGCAGAACCCTTGTCCTTAGTTGCC-3’ (*Pst* I) / deletion |
|  | F: 5’-CCCAAGCTTTCTATGGCCAATCTTTCC-3’ (*Hind* III),  R: 5’-GGGGTACCTCAGAGATTCATACCGGC-3’ (*Kpn* I) / complemention |
|  | F: 5’-ACTACATTCAGAGTGGTGGGTC-3’  R: 5’-ATTGAGTGAAAAGACTTCGGGT-3’ / RT-qPCR |
| *trans198* | F: 5’-TGCCCGTTCGAGCCAGAGA-3’  R: 5’-GCAAATCAGCTCACTTAATTCGC-3’ / RT-qPCR |
| *trans202* | F: 5’-CACCAGCATCGCCAACACATAG-3’  R: 5’-TTGGCCTGGTTGCTCACTCAGT-3’ / RT-qPCR |
| *trans2192* | F: 5’-GAGACCTTCGGCCTGCCA-3’  R: 5’-CTCCGACGATTGACTGCGG-3’ / RT-qPCR |
| *trans238* | F: 5’-CGCAGACAAAGCCGCCAG-3’  R: 5’-GAAAGGGCCAGCAAGGCAT-3’ / RT-qPCR |
| *trans1513* | F: 5’-CGAGCACGTCTGGACACTGCT-3’  R: 5’-CATCGCGTTGCAGGTAAGTGG-3’ / RT-qPCR |
| *trans3288* | F: 5’-CTGCAGAAGTTCAACCACTGACAAC-3’  R: 5’-GGCGCTTATCGAAGCATCGC-3’ / RT-qPCR |
| *trans3747* | F: 5’-AGCAGGCGCAGCTGGTCG-3’  R: 5’-ACTCCACACACAGCGGCACC-3’ / RT-qPCR |
| *cis3128* | F: 5’-AGCATGGCTGCAAATGGC-3’  R: 5’-GACTTGGCCCAACGGTTAAT-3’ / RT-qPCR |
| *cis954* | F: 5’-CTTTGGCAACTGGCATACGGTG-3’  R: 5’-CTGGCAGGTCTGCAATTGGG-3’ / RT-qPCR |
| *pthXo1*  (AY495676) | F: 5’-ATGGATCCCATTCGTTCGCG-3’  R: 5’-GATCGTCCCTCCGACTGAGCC-3’ / coding sequence cloning by PCR |
| *cya*  (Y00545) | F: 5’-ATGCAGCAATCGCATCAGGC-3’  R: 5’-TTAGCTGTCATAGCCGGAATC-3’ / coding sequence cloning by PCR |
| *hrpG* | F: 5’-CGCAATGTCTCGGTGTTC-3’  R: 5’-GCTGAGTTGCTGCGTTTCC-3’ / RT-qPCR |
| *hrpX* | F: 5’-GCGTATCGGCAGGCTTTGA-3’  R: 5’-GCCAATCGGAAGCACCACTCT-3’ / RT-qPCR |
| *16S rRNA* | F: 5’-TTCATGGAGTCGAGTTGCAG-3’  R: 5’-GTCAAGTCATCATGGCCCTT-3’ / RT-qPCR |
